# Fabrication of Air Cavity Structures Using DRIE for Acoustic Signal Confinement in FBAR Devices

**DOI:** 10.3390/mi16060647

**Published:** 2025-05-29

**Authors:** Raju Patel, Manoj Singh Adhikari, Deepak Bansal, Tanmoy Majumder

**Affiliations:** 1 School of Electronics Engineering, Vellore Institute of Technology (VIT), Chennai 600127, Tamil Nadu, India; 2 School of Electronics & Electrical Engineering, Lovely Professional University, Phagwara 144411, Punjab, India; manoj.space99@gmail.com; 3 Semiconductor Process Technology Group, CSIR-Central Electronics Engineering Research Institute (CEERI), Pilani 333031, Rajasthan, India; deepak@ceeri.res.in

**Keywords:** film bulk acoustic resonator, ZnO, DRIE, acoustic signal confinement, RF measurement

## Abstract

Acoustic energy penetrates into the Si substrate at cavity boundaries. Due to this, the air cavity-based bulk acoustic resonators experience higher harmonic mode, parasitic resonance, and spurious mode. To overcome these effects and enhance the performance parameters, a backside air cavity is fabricated using the deep reactive ion etching (DRIE) method. The DRIE method helps to achieve the optimized active area of the resonator. SiO_2_ film on a silicon substrate as the support layer and ZnO as the piezoelectric (PZE) film are used for the resonator. The crystal growth and surface morphology of ZnO film were investigated with X-ray diffraction, scanning electron microscopy, and atomic force microscopy. FBAR is modeled in a 1-D modified Butterworth–Van Dyke (mBVD) equivalent circuit. As RF measurement results, we successfully demonstrated a FBAR with optimized active area of 320 × 320 μm^2^, center frequency of 1.261 GHz, having a quality factor of 583.8. Overall, this suppression of higher harmonic mode shows the great potential for improving the selectivity of the sensor and also in RF filter design applications. This integration of DRIE-based cavity formation with ZnO-based FBAR architecture not only enables compact design but also effectively suppresses spurious and higher-order modes, which demonstrates a performance-enhancing fabrication strategy not fully explored in the current literature.

## 1. Introduction

Film bulk acoustic wave resonators (FBARs) are key components in the field of RF communications, specifically above 1 GHz frequencies, that deliver better performance with miniaturized size at low cost. Such FBARs, with high *Q*-factors, have use in the growing field of mobile communications as filters, duplexers, and frequency regulators. They can also be used for physical, chemical, and biological sensing applications such as UV light, humidity, temperature, pressure, organic gas, toxic ions, VOCs, explosives, antigens, and proteins [1,2,3,4]. Surface acoustic wave (SAW) devices are widely used in electronics [5], telecommunications [6], sensing [7], and emerging areas such as magnon microwave antennas and reservoir computing [8,9]. Also, they heat up, as the comb electrodes offer high resistance. The resonant frequencies thereby have a serious dependency on the variation of electrode temperature. The resonance mode of FBARs is generated via the thickness extension (TE) vibration of the piezoelectric (PZE) film sandwiched between the top and bottom metal electrodes. The BAW resonators that have PZE layers sandwiched between metal electrodes experience relatively low stress, which results in less heating and thereby offers a better power handling performance. The SAW resonators fabricated with the MEMS technologies have compatibility issues during integration with the traditional technologies, while the BAW resonators are compatible with the same, which aids in reducing the device size and costs [10,11,12,13].

Acoustic resonators play a vital role in numerous widespread applications because of their compact, miniaturized size, high resolution, accuracy, and excellent frequency stability. With the leverage of resonators, there has been rapid growth in sensing technologies, which have been key to obtaining solutions for critical challenges across real-time applications like healthcare, environmental monitoring, and industrial automation. Most importantly, liquid sensing is the key for applications like real-time diagnostics, pollution detection, and chemical process control. Physical sensing: For sensing applications, the resonators must have high sensitivity, selectivity, trace-level detection, and tunability. These requirements make the FBAR a suitable choice for sensing applications, which can have a resonant frequency of 10 GHz [14,15,16].

Furthermore, advancements in sealed-cavity bulk acoustic resonators (SCBARs) provide improved performance in terms of lower energy loss and higher resonance stability. Higher-order mode resonance has been identified as a method of improving selectivity and sensitivity in different applications. Apart from conventional fabrication, new processes such as pulsed laser deposition (PLD) and metalorganic chemical vapor deposition (MOCVD) for AlN thin-film growth have been found to improve FBAR performance. Uniformity of AlN film depositions is critical to frequency response and power capability, and so, accurate fabrication techniques are required. Environmental ruggedness and stability are provided by encapsulation of FBAR devices to enable uniform performance. ScAlN and composite structures are also being demanded as other piezoelectric materials for enhanced future performance. Integrating FBARs with CMOS technology supports miniaturized RF circuit design, offering scalability and reducing fabrication costs. Finally, energy loss mechanisms in FBARs like phonon interactions, dielectric losses, and thermal effects, require further study to optimize device performance [17,18,19].

In recent years, researchers have proposed many methods to develop a high-sensitivity, high-tunability, and high-quality-factor FBAR for various applications with various piezoelectric materials. Singh et al. [20] fabricated a MEMS-based, highly tunable film bulk acoustic resonator (TFBAR) using magnetostrictive (MS) Fe_65_Co_35_ for magnetic field sensing applications. Thin films in the acoustic layer stack. Zhao et al. [21] reported a highly improved quality factor average of 2575 ± 136 of the film bulk acoustic wave resonator by introducing a high-quality ZnO buffer layer. Chen et al. [22] proposed a low-temperature, staged deposition method to address the issue of poor crystal quality of AlScN films in the development of broadband film bulk acoustic resonator (FBAR) filters. To improve the performance of FBAR devices, Cui et al. [23] reported a study on suppressing the parasitic resonance of film bulk acoustic wave resonators. These works developed and found a way to improve the properties of FBAR for high-frequency applications such as sensing applications.

Liquid-phase sensing poses significant challenges due to the strong damping of acoustic waves in liquids. Physical sensing (like VOC, UV, temperature, pressure, and gas) and RF filter applications (like filters and duplexers) have been possible using the thickness longitudinal mode (TLM) of the FBAR. Rughoobur et al. [24] fabricated a resonator in which a carbon nanotube isolation layer is used on top of the active area for enhancing in-liquid quality factor gravimetric sensing. This CNT layer decouples the resonance from direct energy dissipation into the liquid while preserving mass sensitivity.

The performance of FBAR-based resonators is often compromised by the generation of lateral acoustic waves. These unwanted waves give rise to spurious and higher-order harmonic modes that interfere with the resonator’s frequency response. In FBAR structures, discontinuities at the electrode edges and boundaries facilitate the excitation of these spurious modes, which can propagate as new lateral resonances. These transverse modes introduce undesired ripples within the passband of the filter and degrade the quality factor by diverting energy away from the primary resonant mode. Various solutions have been proposed to eliminate or suppress spurious and higher-order harmonic modes in FBAR devices through finite element analysis (FEA) simulations. These techniques include apodization, the use of frame-like structures, incorporation of Bragg reflectors within periodic gratings, implementation of step-like cavities, application of damping materials, and the addition of thickened edge loads on the top electrodes [25,26,27,28,29].

To date, no single technique reported in the literature has been able to simultaneously suppress both spurious and higher-order harmonic modes in FBAR devices. In our previous work, we introduced an active area optimization technique that effectively mitigates these unwanted modes by confining the acoustic energy to the central region of the resonator during operation. This approach offers a significant performance enhancement without introducing additional complexity to the fabrication process [19]. In the present study, we further investigate the fabrication and characterization of the optimized FBAR structure to validate and improve its overall performance.

The MEMS-based fabrication of air-cavity FBARs involves a critical step of forming the bulk cavity using the TMAH wet-etching process. Handling the silicon wafer after complete removal of the silicon beneath the active area is particularly challenging. During the extraction of the wafer from the TMAH bath, there is a risk of the etchant coming into contact with the active device area, potentially damaging the active layers and rendering many devices non-functional. Additionally, the cavity can be punctured due to liquid loading during wafer removal, further reducing yield. Residual silicon beneath the active area can also lead to acoustic energy leakage into the substrate, which suppresses the resonant mode. To address these issues, an alternative approach involves leaving a 20–30 µm thick silicon layer under the active region and removing it later using deep reactive ion etching (DRIE). However, since the sidewalls of the backside cavity are not protected by a masking layer after TMAH etching, vertical etching during the DRIE process can enlarge the cavity dimensions. As a result, the intended active area dimensions (320 × 320 µm^2^) are not precisely achieved, leading to the generation of spurious and higher-order harmonic modes in the resonator’s frequency response. This DRIE method provides a perfect active area as compared to the TMAH method used for the wet etching of silicon substrate [30,31,32].

Four basic FBAR device structures exist: back-trench membrane (cavity) [20], solid-mounted resonator (SMR) [11], polymer as a substrate or a polymer layer as a mirror [14], and a membrane over an air gap [27]. In the first and last structures, the acoustic signal is perfectly confined within the PZE film due to impedance mismatch at active layer–air interfaces. In the second structure, acoustic signal confinement is achieved through introducing a mirror between the bottom electrode and the substrate. In the third structure, the polymer works as a mirror for the acoustic signal and hence, confinement is achieved. A resonator structure is fabricated on a deferred membrane, in general, of SiO_2_ or Si_3_N_4_ in the first and last devices. Anisotropic wet-etching or deep reactive ion etching (DRIE) is used to etch the Si bulk material underneath the active area.

This study presents the fabrication and characterization of FBARs aimed at performance enhancement through acoustic confinement. A fabrication method for an air cavity-based acoustic resonator using DRIE has been discussed. For achieving high-performance parameters, there is a need to reduce the acoustic energy loss due to penetration of it into the substrate, and growth of a ZnO film as a piezoelectric layer must be 002-oriented. The detailed crystal growth and surface morphology of ZnO film were investigated. RF characterization of the resonator was performed using an Anritsu MS2028C vector network analyzer, Atsugi-shi, Japan.

The novelty of this work lies in the implementation of a DRIE-based backside air cavity etching method, which enables precise control of the active area, leading to suppression of spurious and higher-order harmonic modes in FBAR devices. While DRIE and ZnO-based FBARs are individually known, their combined use in this context, specifically to address the challenges posed by wet etching (TMAH), such as layer damage, cavity distortion, and reduced fabrication yield, has not been fully demonstrated [32]. Our approach enables reliable fabrication of CMOS-compatible FBAR devices with improved acoustic confinement and a clean frequency response. Furthermore, this work builds on our previous research in active area optimization, validating the proposed structure experimentally. This addresses a key fabrication bottleneck in FBAR production by eliminating post-TMAH damage risks and ensuring better yield.

## 2. Device Fabrication

FBARs utilize a film of PZE material between the top and bottom electrodes through which excitation is provided. The electrical signal produced as a result of the piezoelectric effect and the acoustic signal produced as a result of the inverse piezoelectric effect are exchanged mutually. A standing wave, in the form of an acoustic signal, is produced by providing an electrical signal. The acoustic signal is back-scattered through the bottom surface and top surface of the ZnO layer–air interface by an impedance mismatch.

**Figure 1:**
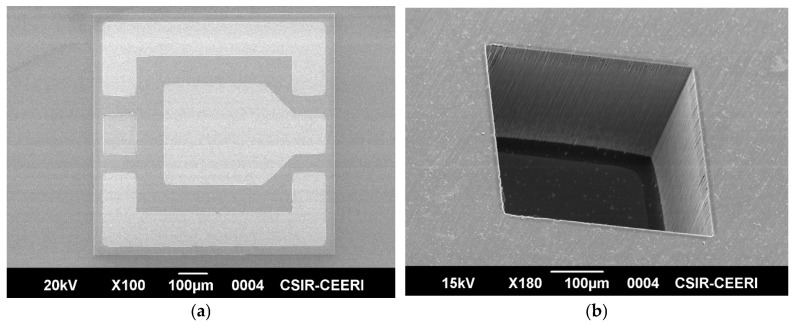
(**a**) SEM image of top view of FBAR. (**b**) Tilted view of backside cavity.

Four photomasks of the FBAR are created with specified requirements and fabricated on a 2″ DSP Si (100) wafer. The process of FBAR begins with degreasing and piranha (H_2_SO_4_:H_2_O_2_ = 3:1) cleaning. The native oxide formed through piranha cleaning has been etched with 5% HF solution, and then the Si wafer is rinsed using DI water. Further, the Si wafer has been dried with an N2 gun and introduced into a thermal oxidation chamber to coat 896 nm SiO_2_. Cr/Au (20 nm/130 nm) as the bottom electrode is sputtered in DC mode. Standard photolithography (SPL) and wet chemical etchant of Au and Cr are employed for patterning the bottom electrode. SPL and wet chemical etching in 1% hydrochloric (HCl) acid solution at room temperature are employed for patterning the PZE layer. The Cr/Au (20 nm/130 nm) top electrode is deposited by DC sputtering and patterned by the liftoff process. SPL is employed to pattern the back-side thermal oxide, and buffered oxide etchant (BOE) is employed to etch the pattern oxide. Deep reactive ion etching (DRIE) is employed for bulk silicon etching to create a backside cavity. Figure 1 is an SEM image of (a) a top view of FBAR and (b) a tilted view of the backside air cavity fabricated using DRIE.

## 3. 1D Equivalent Circuit Modeling

**Figure 2:**
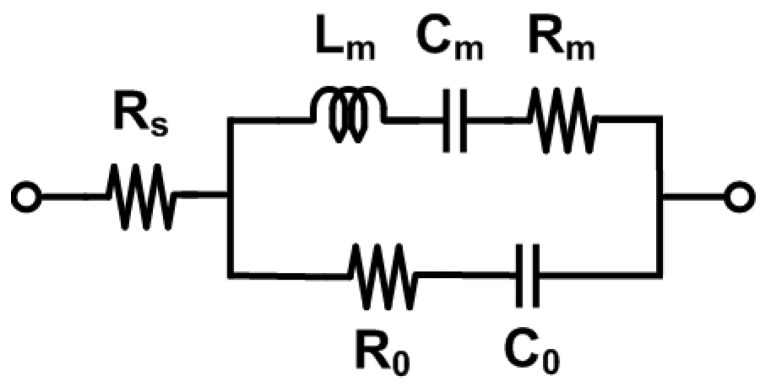
Modified BVD equivalent model of FBAR.

To interpret the measured resonance characteristics of the fabricated FBAR, we employ an electrical equivalent model based on the modified Butterworth–Van Dyke (mBVD) circuit. FBARs are passive devices that can be modeled using a series configuration of capacitor, inductor, and resistor components. This coupled electromechanical model of the FBAR, represented by an electrical equivalent circuit as shown in Figure 2, is called the modified Butterworth Van Dyke (mBVD) model. Here *R*_0_, *C*_0_, *L_m_*, *C_m_*, *R_m_*, and *R_s_* are the FBAR resistance due to medium loss, clamped capacitance between electrodes, dynamic inductance, dynamic capacitance, resistance due to mechanical loss, and lead series resistance, respectively [33,34].

Series resonance frequency $f_{s}$ and parallel resonance frequency $f_{p}$ are determined by

(1) $$f_{s}=\frac{1}{2\pi \sqrt{L_{m}C_{m}}}$$

(2) $$f_{p}=\frac{1}{2\pi \sqrt{L_{m}\frac{C_{m}C_{0}}{C_{m}+C_{0}}}}$$

The elements of the equivalent circuit related to the physical parameters of the resonator are given by

(3) $$k_{eff}^{2}={\left(\frac{\pi }{2}\right)}^{2}\left(\frac{f_{p}-f_{s}}{f_{p}}\right)$$

(4) $$C_{0}=\varepsilon_{0}\varepsilon_{r}\left(\frac{A}{t_{p}}\right)$$

(5) $$C_{m}=C_{0}\left[{\left(\frac{f_{p}}{f_{s}}\right)}^{2}-1\right]$$

(6) $$L_{m}=\frac{1}{C_{m}{\left(2\pi f_{s}\right)}^{2}}$$

(7) $$R_{m}=\frac{2\pi L_{m}f_{s}}{Q_{s}}$$

(8) $$R_{0}=\frac{1}{2\pi C_{0}f_{p}Q_{p}}$$

(9) $$R_{s}=\frac{R_{∎}L}{w}$$

where *ε*_0_ is the permittivity of free space; *ε_r_* is the relative permittivity of the piezoelectric material; A is the active area of the resonator; *t_p_* is the thickness of the piezoelectric layer; *Q_s_* and *Q_p_* are the quality factors at series and parallel resonant frequency, respectively; $R_{∎}$ denotes the series resistance that accounts for the electrical losses originating from the electrode, also known as the square resistance of the top electrode; *L* and *w* are the length and width of the top electrode.

The quality factor *Q* can be calculated by using Equation (10):

(10) $$Q_{f_{x}}=\frac{f_{x}}{2}{\left|\frac{d\varphi_{z}}{df}\right|}_{f_{x}}$$

where $\;f_{x}$ corresponds to either series resonance frequency $\;f_{s}$ or parallel resonance frequency $\;f_{p}$ of the FBAR. The performance parameters of an FBAR are measured in the form of the figure of merit (*FoM*). It is calculated by the relation [27]:

(11) $$FoM=k_{eff}^{2}\times Q$$

## 4. Results and Discussion

### 4.1. Structural and Surface Morphology Study

**Figure 3:**
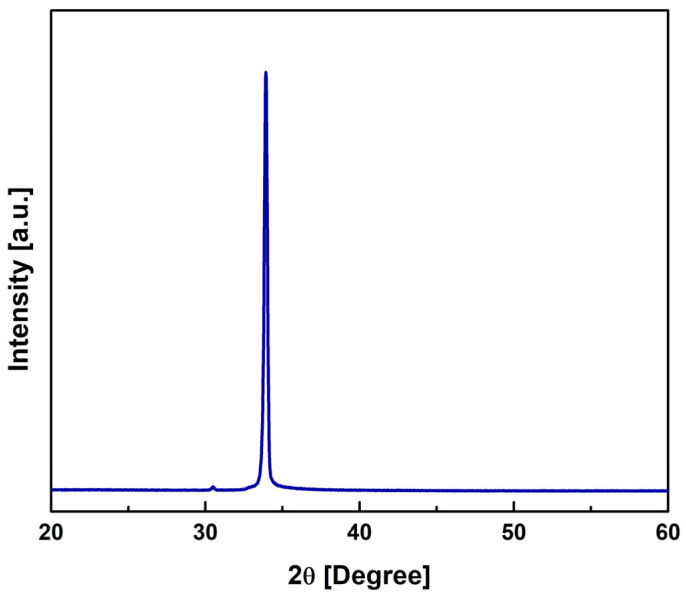
The θ–2θ X-ray scans of the deposited ZnO film.

The structural and surface morphology of ZnO film were investigated with X-ray diffraction (XRD), scanning electron microscopy (SEM), and atomic force microscopy (AFM). Figure 3 depicts the XRD plot of ZnO film deposited on Au/Si substrate. The XRD plot clearly reveals the formation of hexagonal wurtzite structure along the (002) crystallographic plane at 2θ = 34.4437° (PDF card: 036-1451). The average crystalline size, measured using the Scherrer formula, is calculated to be 45.9 nm for the (002) crystallographic plane.

**Figure 4:**
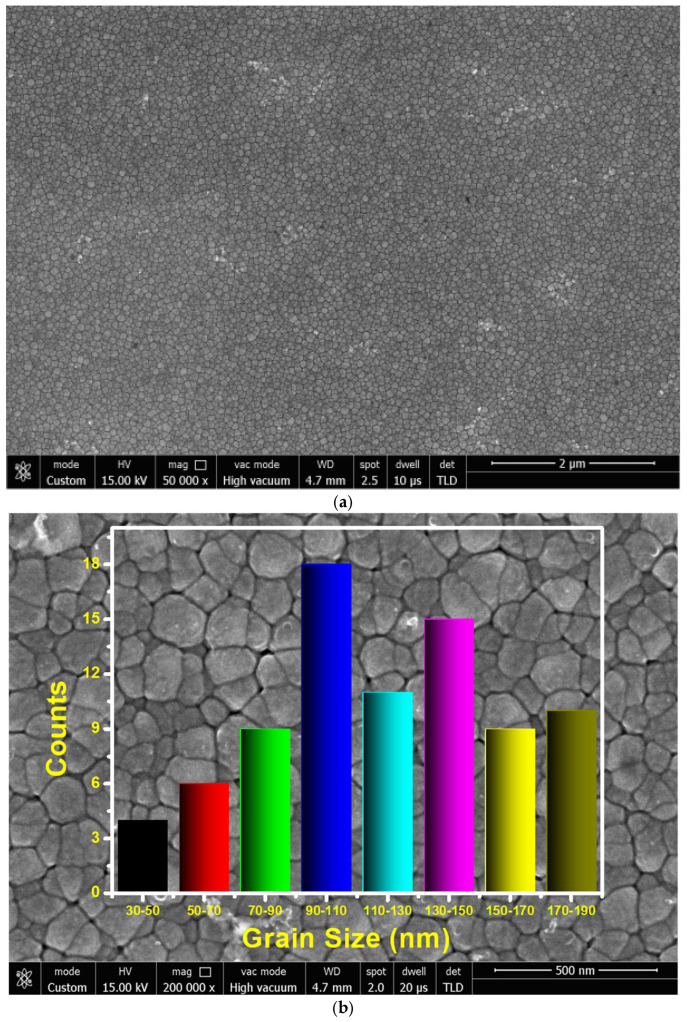
(**a**) SEM micrograph of the PZE film. (**b**) Grain size distribution histogram with corresponding SEM image of the PZE film.

The SEM micrograph of the ZnO film, as shown in Figure 4a,b, shows the grain size distribution histogram with corresponding SEM image. As shown in Figure 4b, grain size was distributed in the range of 31.86 nm to 188.21 nm, with an average size of 118.22 nm. It can be confirmed from the SEM image that the ZnO surface was smooth and the grains were uniformly distributed over the bottom electrode with good adhesion. The morphology of the ZnO film grains was found to be continuous and dense over the substrate.

**Figure 5:**
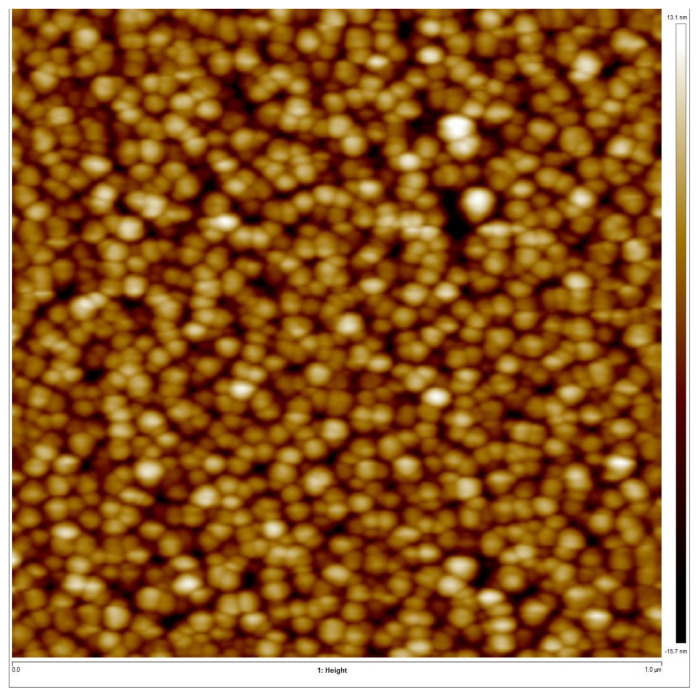
2-D AFM image of the ZnO film surface over an area of 1 × 1 μm^2^.

**Figure 6:**
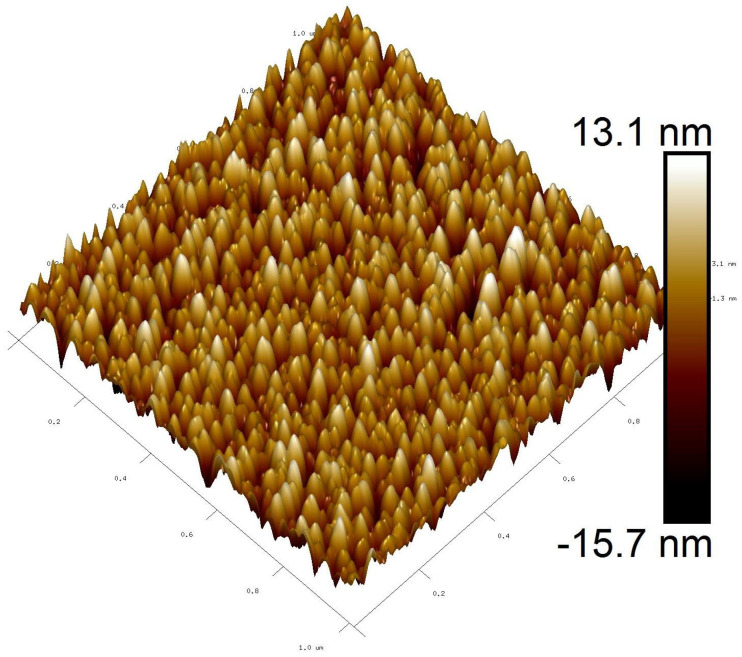
3-D AFM image of the ZnO film over an area of 1 × 1 μm^2^.

**Figure 7:**
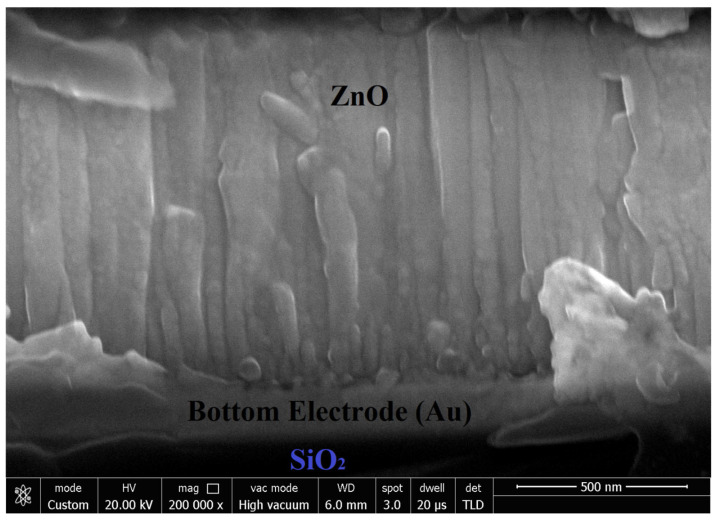
Cross-sectional FESEM image of the ZnO layer.

AFM characterization was used to study the surface roughness of the deposited ZnO film. The 2-D and 3-D AFM images of the ZnO film are represented in Figure 5 and Figure 6, respectively. The AFM images reflect that the ZnO surface was soundly polished. Surface roughness was measured to be 7.45 nm over the area of 1 × 1 μm^2^. A cross-sectional FESEM image of the ZnO layer, as shown in Figure 7, also confirms the C-axis-oriented growth of the ZnO layer with a thickness of 1.5 µm.

### 4.2. RF Characterization of FBAR

**Figure 8:**
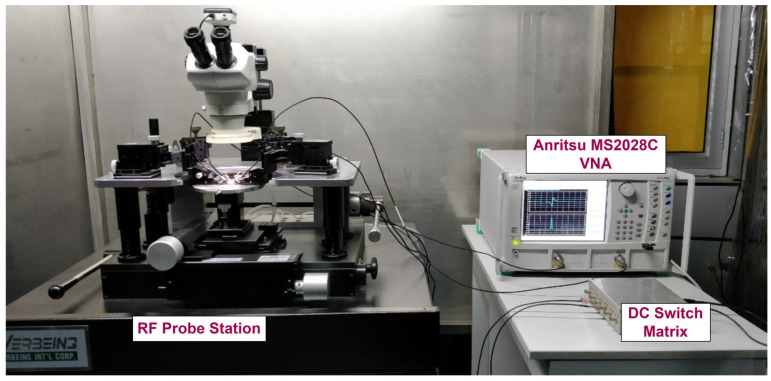
RF measurement setup with VNA.

The RF characterization in the provided result for the built FBAR was performed using an RF probe station in GSG-250 setup combined with the Anritsu MS2028C vector network analyzer (VNA). The RF measurement setup of VNA is depicted in Figure 8. The manufactured ZnO-based resonator from the four-layer (SiO_2_/Au/ZnO/Au) combined structure had optimal device dimensions of size 320 × 320 µm^2^ as the active area and a backside cavity of size 300 × 300 µm^2^. The two-port RF probes were calibrated using Agilent CS-10 substrate, short, open, and load tests. This calibration process shifts the measurement plane onto the RF (GSG-250) probe tip rather than from the VNA. After calibration, loss of −0.1 dB is observed as an error bar.

**Figure 9:**
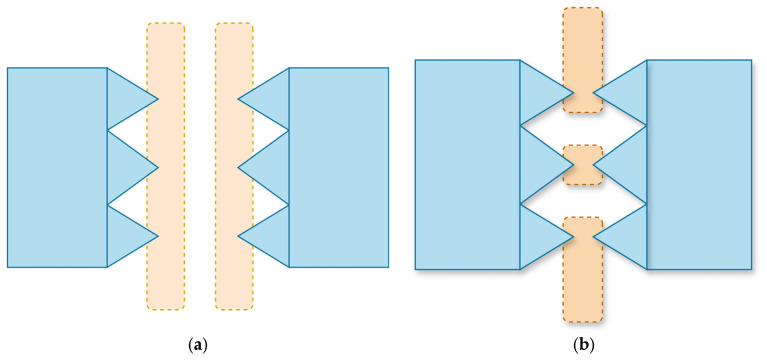
Schematic diagram for the on-wafer: (**a**) short calibration and (**b**) open calibration.

To mitigate parasitic effects, on-wafer short and open calibration keys were measured under the identical conditions as the device under test and are illustrated in Figure 9. This approach effectively removes the impact of all parasitics, with the exception of substrate loss and the inductance associated with metal interconnects.

**Figure 10:**
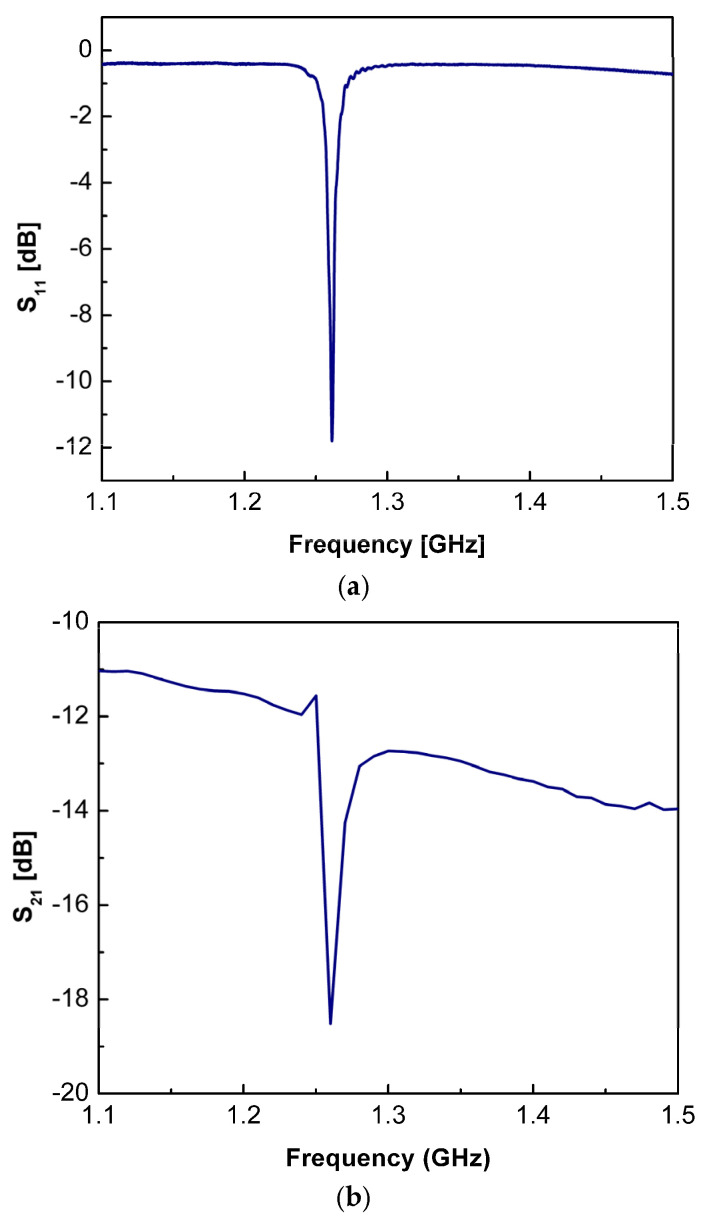
(**a**) The measured reflection spectra (S_11_) as a function of frequency. (**b**) The measured transmission spectra (S_21_) as a function of frequency.

The above device reflection, transmission, impedance/admittance and their phase spectra were subsequently plotted using the derived.csv file. Figure 10a,b illustrates evaluated reflection spectra S_11_ and the transmission spectra S_21_, accordingly, versus frequency. It shows a return loss of −11.8 dB at a given central frequency of 1.261, GHz with a quality factor of 583.8.

**Figure 11:**
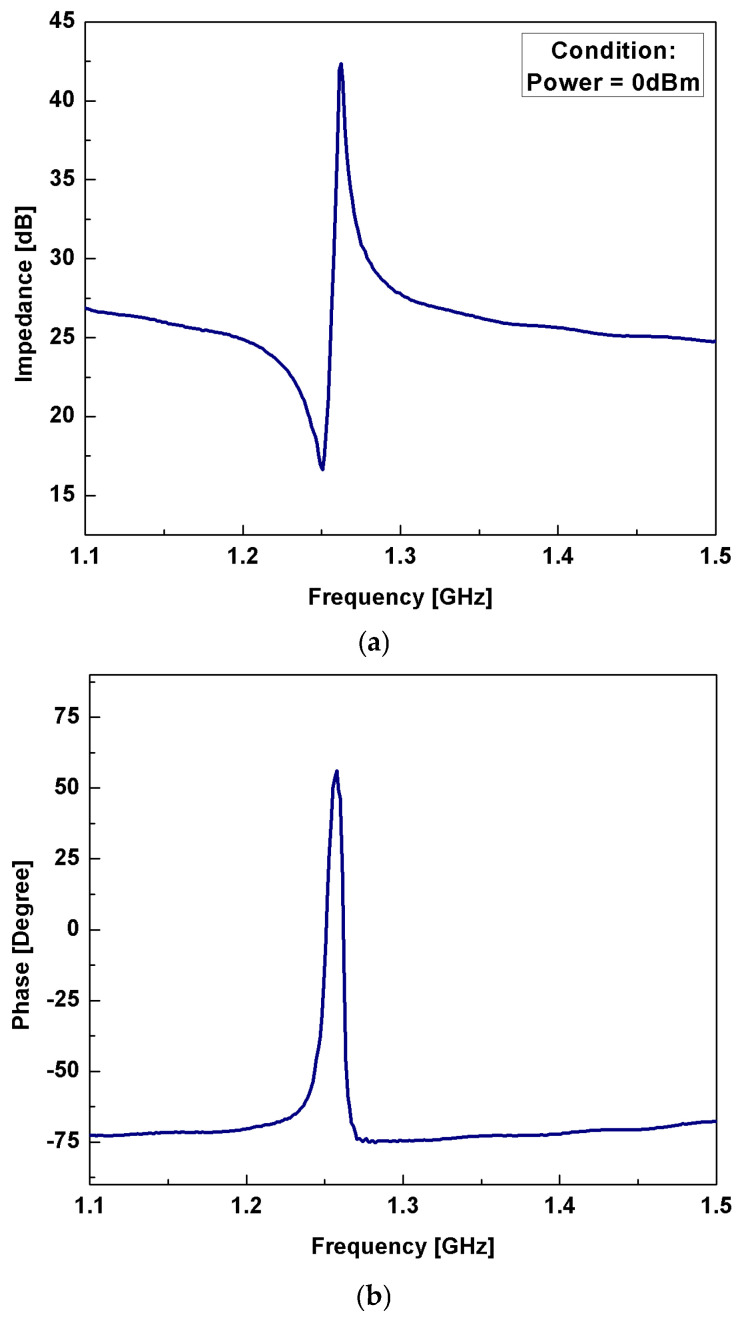
(**a**) The evaluated impedance spectra as a function of frequency. (**b**) The evaluated phase spectra as a function of frequency.

Figure 11a,b illustrates the measured impedance spectra and the phase spectra, respectively, versus frequency. Then, the performance parameters of the resonator are derived from the impedance response. From the response, series resonance frequency *f_s_* is determined to be 1.251 GHz with the lowest impedance value of 16.62 dB, and parallel resonance frequency *f_p_* is determined to be 1.262 GHz, with the highest impedance value of 42.36 dB.

The efficient electromechanical coupling coefficient $k_{eff}^{2}$ and quality factors *Q* collectively are the main performance limitations for FBAR. Additionally, the $k_{eff}^{2}$ defines the bandwidth of an FBAR-based filter that is the parallel resonance frequency *f_p_* minus the series resonance frequency *f_s_*. The value of the *Q* factor that is high defines the sharp slope of an FBAR-based filter. The efficient electromechanical coupling coefficient $k_{eff}^{2}$ was found to be 2.3%. The quality factors (*Q_s_* and *Q_p_*) at series and parallel resonant frequencies of the FBAR were 206 and 353, respectively.

The value of clamped capacitance, measured using an Agilent (E4980A) precision LCR meter, Santa Clara, CA, USA, was about 11.87 pF. Using the mBVD model with lead series resistance R_s_, resistance due to medium loss R_0_, and motional arm component (*L_m_*, *C_m_*, and *R_m_*) values were evaluated. The extracted component values of the mBVD model for the FBAR were *C*_0_ = 11.87 pF, *C_m_* = 0.225 pF, *L_m_* = 0.072 µH, *R_m_* = 2.746 Ω, *R*_0_ = 0.03 Ω, and *R_s_* = 2.3 Ω.

Lastly, Table 1 illustrates the comparison of the fabricated FBAR performance parameters with state-of-the-art devices. It evidently indicates that the fabricated FBAR has improved performance, thus becoming a promising candidate for forthcoming RF filters and sensing purposes. While some prior works demonstrated higher individual *Q* factors or coupling coefficients, they often relied on complex piezoelectric materials (e.g., ScAlN) or multi-step fabrication schemes. Our method strikes a balance between performance and fabrication simplicity. The DRIE-based approach enables improved confinement of acoustic energy, resulting in clean resonance characteristics, reduced spurious activity, and reproducibility across devices, all achieved using standard ZnO layers and CMOS-compatible processes. These advantages make the proposed method a promising candidate for RF filter and sensing applications where manufacturing robustness and frequency purity are critical.

The proposed fabrication process offers an efficient approach without the inclusion of any additional lithography steps and requires only modification of the top electrode mask, while the utilization of DRIE for air cavity formation introduces higher processing costs as compared to wet etching. This fabrication technique provides superior control and precision cavity size that may justify the additional cost depending on application requirements. This trade-off between performance and cost signifies a key consideration in the RF filter and physical sensing application of the proposed architecture.

To conclude the performance discussion, it is worth noting here that although this work focuses primarily on the fabrication and characterization of FBAR structures, the measured performance parameters (resonant frequency of 1.251 GHz, *Q* factor of 353, and coupling coefficient of 2.3%) fall within the typical range suitable for RF filter [22,39,40] and sensing applications [3,14,16]. Such values suggest strong potential for direct integration into front-end modules for wireless communication systems or as a sensing element for gas or VOC detection platforms.

## 5. Conclusions

The active area of the FBAR plays a significant role in improving performance characteristics. This active area is perfectly obtained by the fabrication of an air cavity using the DRIE method. In this work, an air cavity-based FBAR was fabricated and the RF performance parameters were measured using VNA. Generation of the longitudinal mode of the resonator was obtained by the (002) crystallographic growth of ZnO film. The DRIE-fabricated air cavity structure enables effective acoustic energy confinement, which contributes to the suppression of higher-order modes, parasitic resonances, and spurious responses. The device achieved a series resonance frequency of 1.251 GHz and a parallel resonance frequency of 1.262 GHz, with an electromechanical coupling factor of 2.3% and quality factors of 206 and 353, respectively. The performance parameters were extracted using a 1-D electrical equivalent mBVD circuit. The figures of merit (*FoM*) were achieved for the *f_s_* and *f_p_* of 4.74 and 8.12, respectively.

## Figures and Tables

**Table 1:** Comparisons of RF performance parameters of fabricated FBAR with state-of-the-art devices. While some devices in the literature show higher individual metrics, the proposed method offers a reliable fabrication pathway with clean frequency response and CMOS compatibility.

References	[35]	[17]	[36]	[20]	[37]	[38]	This Work
PZE material	AlN	Al_0.88_Sc_0.12_N	ZnO	ZnO	ZnO	ZnO	ZnO
$t_{PZE}$ [μm]	0.461	1	0.65	2.4	2	1	1.5
$f_{p}$ [GHz]	6.84	1.58	1.85	0.879	1.44	2.127	1.251
$k_{eff}^{2}$ [%]	5.64	1.47	2	0.84	7.5	2.03	2.3
*Q*	135	454	350	740.74	115	253.2	353
*FoM*	7.61	6.67	7	6.22	8.62	5.35	8.12

## Data Availability

The original contributions presented in this study are included in the article. Further inquiries can be directed to the corresponding author(s).

## Abbreviations

The following abbreviations are used in this manuscript:

DRIE	Deep reactive ion etching
PZE	Piezoelectric
ZnO	Zinc oxide
RF	Radio frequency
VOC	Volatile organic compounds
IDT	interdigital transducer
mBVD	Modified Butterworth–Van Dyke
TE	Thickness extension
SAW	Surface acoustic wave
FBAR	Film bulk acoustic resonator
PLD	pulsed laser deposition
MOCVD	Metalorganic chemical vapor deposition
TLM	Thickness longitudinal mode
TSM	Thickness shear mode
